# Risk factors for hospitalizations associated with depression among women during the years around a birth: a retrospective cohort study

**DOI:** 10.23889/ijpds.v4i1.453

**Published:** 2019-01-21

**Authors:** Jennifer Christine Fairthorne, Gillian E Hanley, Rollin Brant, Tim F Oberlander

**Affiliations:** 1 British Columbia Children’s Hospital Research Institute, 938 West 28th Avenue, Vancouver, BC Canada V5Z 4H4; 2 Telethon Kids Institute, University of Western Australia; 3 Department of Obstetrics and Gynaecology, University of British Columbia, Faculty of Medicine, 1125 Howe Street, Vancouver, BC Canada V6Z 2K8; 4 Department of Statistics, University of British Columbia

**Keywords:** Mothers, Socio-economic status, Antidepressive agents, Pregnancy, Post-partum period, Parturition, Prescriptions, Hospitalization

## Abstract

**Introduction:**

Socio-economic status (SES) is an important determinant of health. Low SES is associated with higher rates of prenatal and post-partum depression, and prenatal and post-partum depression are associated with sub-optimal maternal and infant health. Furthermore, increased negative effects of post-partum depression have been reported in children from low SES backgrounds.

**Objective:**

To assess whether SES was related to the risk of a medical or psychiatric hospitalization associated with depression (HAWD) and the risk of a HAWD by anti-depressant (AD) use during the years around a birth.

**Methods:**

This retrospective cohort study used linked birth, hospitalization, prescription and tax-file records of the study cohort. We linked registry data of 243,933 women delivering 348,273 live infants in British Columbia (1999-2009). The outcomes of interest were a HAWD and a HAWD with the associated patient AD use. Ranked area-based measures of equivalised, family disposable income were used to create income deciles, our proxy for SES. Decile-1 represented the lowest income areas, and mothers from Decile-6 (middle-income) were the comparator group. Anti-depressant use was defined as having a prescription for a selective serotonin reuptake inhibitor (SSRI) or other AD during the years around a birth, defined as the period beginning 12 months before conception and ending 12 months after the birth. We analysed by pregnancy using mixed effects logistic regression whilst adjusting for maternal age and parity.

**Results:**

Compared to mothers from middle-income areas (Decile-6), mothers from low income areas (Decile-1, Decile-2) had increased odds of a HAWD [adjusted OR=1.77 (CI: 1.43, 2.19); adjusted OR=1.56 (CI: 1.26, 1.94)]. Mothers from low income areas with depression and no AD use had even higher odds of a HAWD [adjusted OR=1.83 (CI: 1.33, 2.20); adjusted OR=1.71(CI: 1.33, 2.20)].

**Conclusions:**

This study provides preliminary evidence to suggest that barriers to treating depression with ADs in mothers from low income areas during the years around a birth might contribute to their increased risk of a HAWD associated with non-pharmacologically treated depression. Further research is needed to understand the reasons for this increased risk.

**Disclaimer:**

All inferences, opinions, and conclusions drawn in this manuscript are those of the authors and do not reflect the opinions or policies of the Data Stewards of Population Data BC.

## Introduction

Socio-economic status (SES) remains one of the most important determinants of health and well-being across the life course (1) and is associated with many health outcomes (2, 3). Yet SES has no recognised definition or gold standard for measurement (3, 4). Some measures of SES, such as the Hollingshead Index of Social Status are structured and are comprised of combinations of material dimensions including income, residential location, occupation and education (5). Government bodies are often reluctant to release individual income data. Instead, researchers must use an area-based proxy for family income. In Canada, area-based measures of equivalised family disposable income have been found suitable for comparing differences in income of more than four deciles (6). Statistics Canada provide a ranking of ten levels of these data by post-code areas of 400 to 700 residences. In keeping with Rossi and Gilmartin’s (7) criteria for a valid and useful social index, the aforementioned equivalised income data are conceptually based (8), valid (9), reliable (9), accessible (10) and complete (8) and are commonly used in health research (9).

In North America, rates of prenatal and post-partum depression were estimated at 12% to 18% (11, 12). In studies that used different proxies for SES (such as education, income, housing conditions and employment), women of lower SES had higher rates of prenatal (13, 14) and post-partum depression (12, 14-16). Despite these statistics, a study of overall health service utilization in 1,000 women in Ontario, Canada (17) found no difference in use between the socially advantaged and disadvantaged during a four week post-partum period. On the other hand, Scottish women from the most deprived quintile had the highest proportion of psychiatric hospitalizations during pre-pregnancy, pregnancy and over a two-year post-partum period (18). Prenatal depression is associated with negative maternal health behaviours such as increased use of non-prescription drugs (19, 20) and negative neonatal and child health outcomes including premature birth (15), lower birth weight (15) and an increased risk of later mental health issues in the child (21, 22). Post-partum depression is associated with poorer developmental trajectories of the child (23) such as poorer language (24) and overall cognitive development (25).

Increased associations of the negative effects of post-partum depression in children from low SES backgrounds have been reported (22, 26, 27). For example, the results of a French cohort study (26) used the responses of nearly 2,000 mothers and indicated that children with mothers of low income with post-partum depression had a significantly higher risk of developing intense, negative, emotional reactions. It is difficult to separate the effects of prenatal anti-depressant (AD) use and maternal depression on the fetus (28) and it is now considered that some previous studies investigating the effect of prenatal AD use may have been confounded by co-existing depression (28). In 2007, a large population-based study (29) accessed registry data of more than one and a half million pregnancies and used innovative statistical methods to allow for possible confounding. While AD use in the first trimester was associated with a slight increase in preterm births, previously documented associations with infants who were small for gestational age (30) and attention-deficit/hyperactivity disorder (31) were not present. This emphasizes that early diagnosis and treatment of depression in women of child-bearing age are important and risks associated with prenatal AD use may be less than previously ascertained. Furthermore, for each patient, the risk of not treating a depressive illness needs to be considered in relation to the risk associated with prenatal treatment with an AD (32).

While relatively little is known regarding the relationship between SES and AD use in the year before conception, during pregnancy and the post-partum, two small studies have reported lower rates of AD use among depressed women of lower SES, as indicated by the proxies of sub-optimal home environment (33) and lower educational attainment (34). If women of lower SES are less likely to use ADs and are less able to access alternative forms of therapy such as cognitive behavioural therapy or counselling (35, 36) due to cost or other issues, we might expect a relationship between lack of access to pharmacotherapy and hospitalizations (medical or psychiatric) associated with depression (HAWDs). This is further supported by several studies, which indicate that a combination of pharmacotherapy with cognitive behavioural therapy or interpersonal therapy results in the best outcomes for women with post-partum depression (37, 38). As a result of the Canada Health Act (39), Canada has a one-tiered health-care system where hospitalizations are free but prescription drugs are not. This suggests that one of the driving factors behind this phenomenon might be the cost of ADs. As a result, provinces have independently developed prescription drug insurance programs and all include some patient co-payment. In British Columbia (BC), the program is entitled Fair PharmaCare and is an income-based program where co-payment increases with rising household income. However, even for those of the lowest household incomes, there is a 30% co-payment rate up to a maximum of 2% of gross household income (40). Further, there is a large body of research indicating that even small co-payments represent access barriers among vulnerable populations (41-44).

In summary, area-based income data provide useful approximations for comparing major differences in income. There are higher rates of prenatal and post-partum depression in women of low SES though this may not be reflected by health service use. Negative associations of maternal depression on the fetus and subsequent child, along with adverse interactions of post-partum depression with low SES and, to a lesser extent, negative effects of prenatal AD use on the resultant child have been implicated. There is also evidence that co-payments associated with ADs provide a barrier to access for those of low SES. Finally, reports of health care use in Canadian women by SES around the time of a birth are scant and inconsistent and we found no studies examining depression and AD use over SES during the years around a birth. Therefore, by using linked registry data, our aim was to explore whether mothers of low SES are at an increased risk of hospitalizations associated with depression and whether this risk is exacerbated in those untreated with an AD.

## Methods

To estimate maternal SES, we used income band data derived from tax-file records for 2002 and 2006 (58). For each post code (comprising 400-700 residences), equivalised family disposable incomes (allowing for the number and age of people within the household) had been calculated, averaged and ranked into ten incremental bands where Decile-1 represented the most disadvantaged mothers. For births after June 30, 2004, we used 2006 data for assessment and otherwise, 2002 data.

By linking registry data during time periods associated with a birth and compared to women from Decile-6, over SES, we aimed to report the odds of:

1. At least one HAWD
2. At least two HAWDs
3. At least one HAWD with a primary diagnosis associated with depression
4. At least one HAWD by AD use

Finally, we aimed to identify linear or quadratic trends over increasing SES for each of the previous outcomes.

Given that women of lower SES have higher rates of prenatal (13, 14) and post-partum depression (12, 14, 16), depression generally (45) and poorer overall health (15), we hypothesized that women of lower SES would have highest odds of each outcome during periods associated with a birth. For most outcomes associated with poorer health, there are negative linear trends over increasing SES (46-48). Therefore, we hypothesized that each of our outcomes would also have a negative linear trend over increasing SES. Due to the lack of available evidence, we were less sure of the relationship of at least one HAWD and AD use over SES. However, since women of lowest SES are less likely to access ADs (33, 34), and more likely to experience prenatal and post-natal depression, and depression generally (13, 14, 16), we hypothesized that they would be more likely to experience a HAWD with no AD use.

After obtaining approval from the Behavioural Research Ethics Board (49) and all relevant data stewards (10), Population Data BC supplied complete, accurate and valid datasets (50-52) from Perinatal Services (53), the Insurance Registry (54), PharmaNet (55) and the Hospital Registry (56) along with Income band data (57) prepared by Statistics Canada.

### Cohort Definition

Our cohort comprised women who gave birth to a live singleton in BC from 1999-2009 inclusive. This included about 95% of all women who lived in BC before, during and after their pregnancies. Women indigenous to Canada or from the Military were excluded since their health insurance was from a different source. All residents of BC are registered for health insurance. Therefore, to ensure that study mothers lived in BC for the majority of the study period, we required that mothers be registered for health insurance for at least 275 days in the calendar year before the infant’s birth, the birth year and the following calendar year.

### Time periods

For each pregnancy, we examined hospitalizations within one or more of the periods: pre-pregnancy (the 12 months before conception), pregnancy, extended post-partum (the 12 months after the birth), and the combined period (the combination of the previous three periods). The date of admission was used to allocate a hospitalization to a time period. We included the combined period in order to utilize the increased power resulting from the greater number of hospitalizations during this longer period. We reasoned that some associations of SES, might be identified during the combined period that were not apparent in the shorter, contributing periods, particularly for less frequently occurring outcomes.

### Protecting respondent privacy

Statistics Canada ensures respondent privacy and confidentiality during the linkage process and subsequent use of linked files. Only employees directly involved in the linkage process have access to the unique identifying information required for linkage (such as names and birthdates) but have no access to the analysis variables. Once the data linkage process is complete, the resulting linked keys are used to create a linked file without identifying information and only the de-identified file is accessed by analysts for research purposes including validation and statistical analyses. The application for the record linkage was reviewed and approved by the Executive Management Board at Statistics Canada under the Statistics Canada Policy on Record Linkage (see http://www.statcan.gc.ca/eng/record/policy4-1.)

### Outcome measures

Each hospitalization had provision for listing a maximum of 25 ICD-9 diagnostic codes, which were provided by the attending clinician. We defined a HAWD as a hospitalization with a listed ICD-9 code of 296.2, 296.3, 296.9, 298.0, 300.4, 309.1 or 311 or an ICD-10 code of F32, F33, F34.1, F34.9, F38, F39 or F43.21. A HAWD with a primary diagnosis associated with depression was defined as one where the first listed ICD-9 or ICD-10 code was associated with depression (as described previously). This is because the first listed diagnostic code in the hospitalization data represents the diagnosis that was primarily responsible for the hospital admission. Our principal outcome measure was at least one HAWD over each of the four defined time periods. Due to smaller numbers of women having at least two HAWDs or at least one HAWD with a primary diagnosis associated with depression, we calculated the odds of at least two HAWDs and at least one HAWD with a primary diagnosis associated with depression only over the combined period. Having at least two HAWDs provided an indication that the depression was recurrent and having at least one HAWD with a primary diagnosis associated with depression indicated that severity of the depression was likely to be more severe than other HAWDs.

### Other covariates and exposures

Since maternal age (59) and parity (60) are related to the risk of depression, we included each trait at the infant’s birth-date as explanatory variables. The variable - *Maternal age* had four categories: *Less than 20 years*, *20-29 years*, *30-39 years* and *40 years or older*. *Parity* had two levels: *Nulliparous* and *Multiparous*. A variable provided by the *Perinatal Data Registry* was *Final gestational age* (in complete weeks) which had been calculated using an algorithm incorporating the last menstrual period, first ultrasound, newborn examination and maternal charts (61). We subtracted *Final gestational age* (in days) from the infant’s date of birth to provide the estimated date of conception.

We defined an AD as either a selective serotonin reuptake inhibitor (SSRI), a serotonin and norepinephrine re-uptake inhibitor (SNRI) or other anti-depressant (OAD) (Supplementary table 1). For each member of the cohort, *AD use* was defined as having filled at least one prescription for an SSRI, SNRI or OAD during the combined period.

### Analyses

For each outcome, mothers with no HAWD formed the reference group and mothers from Decile-6 were the comparator group. We chose a middle decile (rather than Decile-1 or Decile-10) as the comparator since we wanted to compare the odds ratios (ORs) of mothers from the lowest income areas (Decile-1) and mothers from the highest income areas (Decile-10). Within the eleven year study period, mothers had varying numbers of pregnancies. To allow for the resulting clusters, we used Mixed Effects Logistic Regression with the variable, *maternal identity number* specifying the random effects and the variable, *pregnancy identity number* specifying the fixed effects. The type or number of HAWDs was the dependent variable and we assessed odds by pregnancy and SES decile. For each outcome over a time period, we used a separate regression model. All unadjusted odds ratios (uORs) and adjusted odds ratios (aORs) are tabulated but we report only significant aORs in the text. In the analyses associated only with HAWDs, we made comparisons over six outcomes (at least one HAWD over each of four time periods, at least two HAWDs over the combined period and a HAWD associated with a primary diagnosis over the combined period) and over nine SES deciles. Hence, using Bonferroni’s approximation for multiple comparisons (62), we divided the usual significance level (0.05) by 54 (6 x 9) to produce a new significance level of 0.001. In our investigation of AD use and HAWDs, we made comparisons over the two outcomes of at least one HAWD with AD use and at least one HAWD without AD use and nine deciles resulting in 18 comparisons. Here, the application of Bonferroni’s approximation produced an adjusted significance level of 0.003 (0.05/18). When investigating the presence of a linear or quadratic trend, we again used *Mixed Effects Logistic Regression*, but, we reverted to the traditional level of significance (0.05) since no comparisons were being made. We compared proportions using a two sample test of proportions which used the *prtesti* command (63). STATA14 was used for all analyses.

## Results

There were 243,933 women with 348,273 pregnancies resulting in live births in BC between January 1, 1999 and December 31, 2009. Mothers from the lowest income areas (Decile-1) were more likely to be of younger age (less than 20 years) compared to mothers from Decile-10 (p-value=0.01). Conversely, mothers from Decile-10 were more likely to be of mature age (40 years or more) than mothers from Decile-1 (p-value=0.001). The proportion of pregnancies associated with each SES decile varied significantly with the largest number, 38,796 in Decile-3 (11.1%) and the smallest, 26,583 (7.6%) in Decile-10 (p-value< 0.005) [Table 1]. Of the pregnancies in our cohort, 383 (0.11%) were subject to at least one HAWD during pre-pregnancy, 293 (0.08%) to a HAWD during pregnancy, 1,551 (0.45%) to a HAWD during the extended post-partum and 2,044 (0.59%) to a HAWD during the combined period. Of the pregnancies associated with a HAWD during the combined period, 433 (21.2%) were associated with AD use and 1,611 (78.8%) were not ( Table 2).

**Table 1: Maternal traits by socio-economic status:** SES, socio-economic status; *, numbers are suppressed in all cells where N<5 due to conditions imposed by the data custodians. Note: Maternal age and parity were calculated at the time of the birth

SES decile	Age in years					Parity		Total
	<20	20-29	30-39	≥40	0	≥1	Missing	

1	1,541	16,769	16,189	1,252	16,506	19,243	*	35,751
	(4.30%)	(46.90%)	(45.30%)	(3.50%)	(46.20%)	(53.80%)		(100%)
2	1,195	17,615	18,430	1,262	17,375	21,123	*	38,502
	(3.10%)	(45.80%)	(47.90%)	(3.30%)	(45.10%)	(54.90%)		(100%)
3	1,111	17,412	18,926	1,347	17,506	21,123	*	38,796
	(2.90%)	(44.90%)	(48.80%)	(3.50%)	(45.10%)	(54.90%)		(100%)
4	970	15,891	18,952	1,259	16,623	20,446	*	37,072
	(2.60%)	(42.90%)	(51.10%)	(3.40%)	(44.80%)	(55.20%)		(100%)
5	895	14,921	19,290	1,400	16,146	20,358	*	36,506
	(2.50%)	(40.90%)	(52.80%)	(3.80%)	(44.20%)	(55.80%)		(100%)
6	851	13,947	19,415	1,420	15,844	19,787	*	35,633
	(2.40%)	(39.10%)	(54.50%)	(4.00%)	(44.50%)	(55.50%)		(100%)
7	721	12,754	19,246	1,437	15,180	18,977	*	34,158
	(2.10%)	(37.30%)	(56.30%)	(4.20%)	(44.40%)	(55.50%)		(100%)
8	666	12,216	19,389	1,428	14,677	19,019	*	33,699
	(2.00%)	(36.30%)	(57.50%)	(4.20%)	(43.60%)	(56.40%)		(100%)
9	646	10,779	18,580	1,568	13,693	17,878	*	31,573
	(2.10%)	(34.10%)	(58.90%)	(5.00%)	(43.40%)	(56.60%)		(100%)
10	449	8,036	16,441	1,657	11,541	15,042	*	26,583
	(1.70%)	(30.20%)	(61.90%)	(6.20%)	(43.40%)	(56.60%)		(100%)

Total	9,045	140,340	184,858	14,030	155,091	193,125	20	348,273
	(2.60%)	(40.30%)	(53.10%)	(4.00%)	(44.50%)	(55.50%)	(0.0%)	(100%)

**Table 2: Numbers and percentages of hospitalizations (medical or psychiatric) associated with a HAWD by SES:** HAWD, hospitalization (medical or psychiatric) associated with depression.

Socio-economic status decile										
1	2	3	4	5	6	7	8	9	10	Total

At least one HAWD during pre-pregnancy										
57	51	46	39	48	33	25	33	26	25	383
0.16%	0.13%	0.12%	0.11%	0.13%	0.09%	0.07%	0.10%	0.08%	0.09%	0.11%
At least one HAWD during pregnancy										
39	41	38	27	34	29	17	26	23	19	293
0.11%	0.11%	10.00%	0.07%	0.09%	0.08%	0.05%	0.08%	0.07%	0.07%	0.08%
At least one HAWD during extended post-partum										
205	222	145	183	154	137	133	133	135	101	1551
0.57%	0.58%	0.37%	0.49%	0.42%	0.38%	0.39%	0.39%	0.43%	0.38%	0.45%
At least one HAWD during combined period										
284	273	211	240	207	171	178	176	169	135	2,044
0.79%	0.71%	0.54%	0.65%	0.57%	0.48%	0.52%	0.52%	1%	0.51%	0.59%
At least two HAWDs during combined period										
47	40	25	30	23	16	13	20	18	7	239
0.13%	0.10%	0.06%	0.08%	0.06%	0.04%	0.04%	0.06%	0.06%	0.03%	0.07%
At least one HAWD with primary diagnosis associated with depression during combined period										
44	46	43	49	39	29	29	28	28	24	359
0.12%	0.12%	0.11%	0.13%	0.11%	0.08%	0.08%	0.08%	0%	0.09%	0.10%
At least one HAWD with anti-depressant use during combined period										
67	55	39	41	29	45	43	40	39	35	433
0.19%	0.14%	0.10%	0.11%	0.08%	0.13%	0.13%	0.12%	0.12%	0.13%	0.12%
At least one HAWD with no anti-depressant use during combined period										
217	218	172	199	178	126	135	136	130	100	1,611
0.61%	0.57%	0.44%	0.54%	0.49%	0.36%	0.40%	0.40%	0.41%	0.38%	0.46%

### Hospitalizations (medical or psychiatric) associated with depression

**Figure 1: Odds of at least two hospitalizations associated with depression over the combined period according to socio-economic status:**
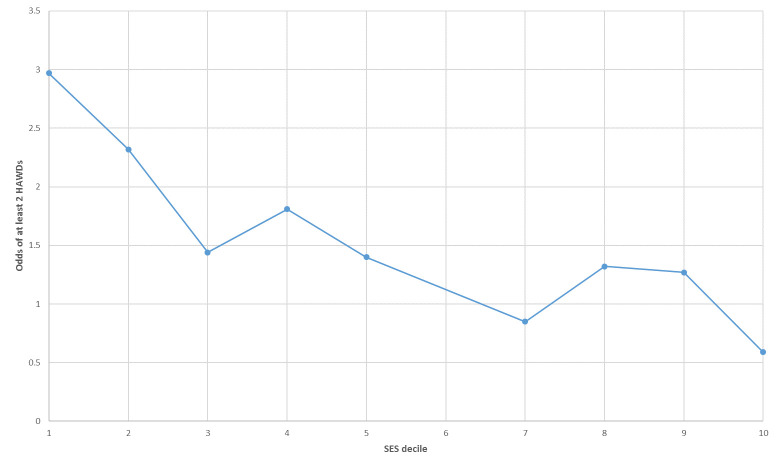
HAWD, Medical or psychiatric hospitalization associated with depression; SES, socio-economic status. Note: Unadjusted odds have been graphed.

During the extended post-partum, compared to mothers from Decile-6, mothers from Decile-1 and Decile-2 had more than 60% increased odds of at least one HAWD [aOR=1.62 (CI: 1.28, 2.07); aOR=1.63 (CI: 1.28, 2.07) p-value <0.001]. During the combined period, compared to mothers from Decile-6, mothers from Decile-1 and Decile-2 had significantly increased odds of a HAWD [1.77 (CI: 1.43, 2.19); aOR=1.56 (CI: 1.26, 1.94) p-values<0.001] (Table 3). During the combined period, compared to mothers from Decile-6, mothers from Decile-1 were nearly three times more likely to have at least two HAWDs [aOR=2.85 (CI: 1.62, 5.03) p-value<0.001]. Further, the odds reduced over increasing SES with a linear trend (p-value<0.001) [Figure 1, Table 4]. There were significant negative linear trends over increasing SES for having a HAWD over all six outcomes with p-values ranging from <0.001 to 0.013 (Supplementary table 2).

**Table 3: Odds (with 95% confidence intervals) of anti-depressant use and hospitalizations (medical or psychiatric) associated with depression over SES:** HAWD, hospitalization (medical or psychiatric) associated with depression; SES, socio-economic status decile; OR, odds ratio; Ref, Reference group; 0, No HAWD from the corresponding SES group over the same time period; primary diagnosis depression, primary diagnosis associated with depression; U, unadjusted OR; A, adjusted OR;. Note 1: Adjusted model allows for maternal age and parity at the birth of the child. Note 2: Level of significance is 0.001 and significant ORs are highlighted.

			At least one HAWD			
SES	Model	Ref (0)	Pre-pregnancy	Pregnancy	Extended post-partum	Combined period

1	U	…	1.72(1.12, 2.65)	1.34(0.83, 2.17)	1.56(1.22, 1.99)	1.74(1.41, 2.15)
	A		1.65(1.07, 2.53)	1.30(0.80, 2.11)	1.62(1.28, 2.07)	1.77(1.43, 2.19)
2	U	…	1.43(0.92, 2.22)	1.31(0.81, 2.11)	1.58(1.24, 2.00)	1.55(1.25, 1.91)
	A		1.38(0.89, 215)	1.28(0.79, 2.06)	1.63(1.28, 2.07)	1.56(1.26, 1.94)
3	U	…	1.28(0.82, 2.00)	1.20(0.74, 1.95)	0.97(0.75, 1.25)	1.15(0.92, 1.43)
	A		1.25(0.80, 1.95)	1.18(0.73, 1.92)	0.99(0.76, 1.29)	1.16(0.92, 1.45)
4	U	…	1.14(0.71, 1.81)	0.89(0.53, 1.51)	1.32(1.03, 1.68)	1.39(1.12, 1.72)
	A		1.12(0.70, 1.78)	0.88(0.52, 1.49)	1.34(1.05, 1.73)	1.40(1.13, 1.74)
5	U	…	1.42(0.91, 2.21)	1.14(0.70, 1.88)	1.11(0.86, 1.43)	1.20(0.96, 1.50)
	A		1.41(0.90, 2.19)	1.14(0.69, 1.87)	1.13(0.87, 1.46)	1.22(0.97, 1.52)
6	Comparator					
7	U	…	0.79(0.47, 1.33)	0.61(0.34, 1.11)	1.04(0.80, 1.35)	1.10(0.87, 1.38)
	A		0.80(0.47, 1.34)	0.62(0.34, 1.12)	1.04(0.80, 1.36)	1.11(0.88, 1.40)
8	U	…	1.06(0.65, 1.71)	0.95(0.56, 1.61)	1.03(0.79, 1.34)	1.10(0.88, 1.39)
	A		1.07(0.66, 1.74)	0.96(0.56, 1.62)	1.03(0.79, 1.35)	1.11(0.88, 1.41)
9	U	…	0.89(0.53, 1.49)	0.90(0.52, 1.55)	1.12(0.86, 1.46)	1.13(0.89, 1.42)
	A		0.91(0.54, 1.52)	0.91(0.53, 1.57)	1.11(0.85, 1.46)	1.14(0.90, 1.44)
10	U	…	1.02(0.60, 1.71)	0.88(0.49, 1.57)	0.99(0.75, 1.32)	1.07(0.83, 1.37)
	A		1.06(0.63, 1.79)	0.91(0.51, 1.62)	0.97(0.73, 1.29)	1.08(0.84, 1.39)

**Table 4: Odds ratios of at least two HAWDs and at least one HAWD associated with a primary diagnosis of depression:** HAWD, hospitalization (medical or psychiatric) associated with depression; SES, socio-economic status decile; OR, odds ratio; 0, No HAWD from the corresponding SES group over the same time period; primary diagnosis depression, primary diagnosis associated with depression; U, unadjusted OR; A, adjusted OR;. Note 1: Adjusted model allows for maternal age and parity at the birth of the child. Note 2: Level of significance is 0.001 and significant ORs are highlighted.

SES	Model	Reference group (0 HAWDs)	≥2 HAWDs Combined period	≥1 HAWD (primary diagnosis depression) Combined period

1	U	…	2.97(1.67, 5.18)	1.49(0.91, 2.43)
	A		2.85(1.62, 5.03)	1.32(0.81, 2.15)
2	U	…	2.32(1.30, 4.14)	1.47(0.91, 2.39)
	A		2.27(1.27, 4.05)	1.36(0.84, 2.20)
3	U	…	1.44(0.77, 2.69)	1.36(0.83, 2.22)
	A		1.41(0.75, 2.64)	1.27(0.78, 2.07)
4	U	…	1.81(0.98, 3.31)	1.64(1.02, 2.65)
	A		1.78(0.97, 3.27)	1.57(0.97, 2.53)
5	U	…	1.40(0.74, 2.66)	1.32(0.80, 2.18)
	A		1.40(0.74, 2.64)	1.29(0.78, 2.12)
6	Comparator			
7	U	…	0.85(0.41, 1.76)	1.05(0.62, 1.79)
	A		0.85(0.41, 1.78)	1.08(0.63, 1.84)
8	U	…	1.32(0.69, 2.55)	1.02(0.60, 1.75)
	A		1.33(0.69, 2.58)	1.05(0.62, 1.80)
9	U	…	1.27(0.65, 2.49)	1.08(0.63, 1.84)
	A		1.29(0.66, 2.53)	1.14(0.67, 1.95)
10	U	…	0.59(0.24, 1.43)	1.11(0.63, 1.94)
	A		0.61(0.25, 1.47)	1.25(0.71, 2.19)

### Anti-depressant use and hospitalizations (medical or psychiatric) associated with depression

During the combined period and compared to mothers from Decile-6, mothers from Decile-1 and Decile-2 were more likely to have at least one HAWD with no AD use [aOR=1.83 (CI: 1.33, 2.20); aOR=1.71 (CI: 1.33, 2.20) p-values<0.0005)]. Likewise, mothers from Decile-4 were more likely to have at least one HAWD with no AD use over the combined period [aOR=1.60(CI: 1.24, 2.06) p-value<0.0005)] See Table 5. In mothers with at least one HAWD and no AD use, there was a negative trend (p-value=0.003) over increasing SES. In mothers with at least one HAWD and AD use, there was a negative trend (p-value=0.001) over increasing SES [Supplementary table 2].

**Table 5: Odds (with 95% confidence intervals) of hospitalizations (medical or psychiatric) associated with depression by anti-depressant use over SES:** HAWD, hospitalization (medical or psychiatric) associated with depression; primary diagnosis depression, primary diagnosis associated with depression; SES, socio-economic status decile; 0, No HAWD from the corresponding SES group over the same time period; U, unadjusted odds ratio; A, adjusted odds ratio. Note 1: Adjusted model allows for maternal age and parity at the birth of the child. Note 2: Level of significance is 0.001 and significant ORs are highlighted.

SES	Model	No HAWD & no AD use	At least one HAWD with no AD use	At least one HAWD with AD use

1	U	…	1.84(1.43, 2.36)	1.60(1.01, 2.52)
	A		1.83(1.33, 2.20)	1.68(1.06, 2.65)
2	U	…	1.71(1.33, 2.19)	1.16(0.72, 1.86)
	A		1.71(1.33, 2.20)	1.20(0.75, 1.93)
3	U	…	1.28(0.99, 1.66)	0.75(0.45, 1.25)
	A		1.28(0.98, 1.66)	0.78(0.47, 1.30)
4	U	…	1.59(1.24, 2.05)	0.86(0.52, 1.42)
	A		1.60(1.24, 2.06)	0.89(0.54, 1.47)
5	U	…	1.44(1.11, 1.86)	0.58(0.33, 1.00)
	A		1.44(1.11, 1.87)	0.59(0.34, 1.03)
6	Comparator			
7	U	…	1.14(0.87, 1.50)	1.00(0.61, 1.66)
	A		1.15(0.87, 1.51)	1.03(0.62, 1.69)
8	U	…	1.17(0.89, 1.54)	0.93(0.56, 1.55)
	A		1.18(0.90, 1.55)	0.95(0.57, 1.57)
9	U	…	1.19(0.91, 1.57)	0.95(0.57, 1.59)
	A		1.20(0.91, 1.58)	0.96(0.58, 1.60)
10	U	…	1.07(0.80, 1.44)	1.06(0.63, 1.80)
	A		1.08(0.81, 1.46)	1.05(0.62, 1.79)

## Discussion

In this retrospective cohort study, the mothers from the lowest income areas (Decile-1 and Decile-2) had highest odds of at least one HAWD during the extended post-partum and the combined period and at least two HAWDS during the combined period. When AD use was included in the analysis, it was shown that mothers from the lowest income areas had significantly heightened odds of a HAWD with no AD use, and that the risk reduced linearly over increasing SES.

### Hospitalizations (medical or psychiatric) associated with depression

As expected, mothers from low income areas had increased likelihood of all outcomes (at least one HAWD over each of the time periods, at least two HAWDs and at least one HAWD with a primary diagnosis of depression over the combined period). In particular, mothers of Decile-1 had nearly three times the risk of multiple HAWDs over the combined period, and significantly increased risks of a HAWD during the extended post-partum period. Combined periods and all outcomes were associated with a significant negative trend over increasing SES. Such a negative trend has not previously been reported. Our results reflect the association of lower SES and increased rates of depression associated with prenatal and post-partum periods previously cited (12-14, 16). Our findings also likely reflect Canada’s one tier system with hospitalizations at no patient cost and provide evidence of equity of access to hospitalizations associated with depression, regardless of SES, in BC. Our results differ from the from the Ontario study (17) which found no difference in health-care use between socially disadvantaged and advantaged women. This might be accounted for by their use of only urban data, compared to our provincial use. Further, the Ontario study used different measures of health-care use such as GP visits rather than hospitalizations.

### Anti-depressant use and hospitalizations (medical or psychiatric) associated with depression

We found no previous studies which compared the risk of a HAWD in women according to AD use in relation to SES. During the combined period, mothers from low income areas (Decile-1 and Decile-2) with no AD use were significantly more likely to be hospitalized than mothers from Decile-6. Others have reported a relationship between prenatal maternal depressive disorder with no pharmacological treatment and low SES (33, 34). However, the larger size of our study enabled us to identify that the risk of pharmacologically untreated depression and a HAWD reduced significantly with increasing SES (Supplementary table 2). The universal free access to hospitalizations combined with the necessary co-payments for ADs could explain why the risk of a hospitalization with pharmacologically untreated depression is highest in mothers from the lowest income areas and reduces with increasing SES.

### Strengths and limitations

Our linkage of registry data enabled us to access records of about 240,000 women increasing the power to assess smaller differences than other studies in the area. No previous studies have compared the risk of a hospitalization in women with pharmacologically treated and untreated depression according to SES. Moreover, no previous studies have established an SES gradient for hospitalizations associated with depression according to whether the depression was pharmacologically treated. Our proxies for depression (HAWDs) and AD use did not rely on recall but were objectively extracted from administrative data.

A limitation was that our measure of SES, equivalised family income by postcode, is a less precise measure of SES than individual income level and did not include education or occupation. In addition, SES information was generally not available for the year of birth of the child. In some instances, where a woman’s income changed between the birth year and either 2002 or 2006, this may have resulted in less precise SES measures. Also, we were unable to adjust for ethnicity or immigration and there is evidence that some demographic groups have a degree of protection from the effects of low SES on depression (64). Having a partner is also a protective factor for depression (65) and we were unable to adjust for marital status.

### Conclusion

During the years around a birth, mothers from low income areas (Decile-1 and Decile-2) had higher odds of HAWDs and the risk reduced linearly over increasing SES. Pharmacologically untreated depression further increased the risk of a HAWD. Our results provide preliminary evidence that barriers to accessing ADs for mothers of lower SES might contribute to their increased risk of pharmacologically untreated depression. Implications for further research include quantitative and qualitative studies investigating women of low SES during the years around a birth who have experienced a HAWD whilst untreated with ADs. This might enable the identification of the driving factor(s) of this phenomenon.

**Supplementary table 1: Serotonin re-uptake inhibitors and other anti-depressants:** HCL, hydrochloride.

Anti-depressant	Generic names

Selective serotonin re-uptake inhibitors	Fluoxetine, citalopram, paroxetine, sertraline, fluvoxamine, escitalopram, duloxetine HCL
Serotonin and norepinephrine re-uptake inhibitors	Venlafaxine HCL, duloxetine HCL, desvenlafaxine succinate
Other anti-depressants	Amitryptaline, amoxapine, clomipramine, desipramine, doxepin, imipramine, nortriptyline, protriptyline, trimipramine, phenelzine, hydroxytryptophan, tranylcypromine

**Supplementary table 2: Linear trends of outcome measures over increasing SES:** ≥, at least; HAWD, hospitalization (medical or psychiatric) associated with depression; AD, anti-depressant (defined in Supplementary table 1). Note 1: Level of significance is 0.05. Note 2: There were no quadratic trends.

Time period	Outcome	Sign and p-value

Pre-pregnancy	≥1 HAWD	Negative, < 0.0005
Pregnancy	≥1 HAWD	Negative, 0.007
Extended post-partum	≥1 HAWD	Negative, < 0.0005
Combined period	≥1 HAWD	Negative, < 0.0005
Combined period	≥2 HAWDs	Negative, < 0.0005
Combined period	≥1 HAWD with primary diagnosis associated with depression	Negative, 0.013
Combined period	≥1 HAWD & no AD use	Negative, 0.003
Combined period	≥1 HAWD & AD use	Negative, 0.001

## Abbreviations

SES	socio-economic status
AD	anti-depressant
CI	confidence interval
HAWD	medical or psychiatric hospitalizations associated with depression
BC	British Columbia
SSRI	selective serotonin reuptake inhibitor
SNRI	serotonin and norepinephrine re-uptake inhibitor
OAD	other anti-depressant
OR	odds ratio
uOR	unadjusted odds ratio
aOR	adjusted odds ratio
